# 4-Allyl-2-methoxyphenol modulates the expression of genes involved in efflux pump, biofilm formation and sterol biosynthesis in azole resistant *Aspergillus fumigatus*

**DOI:** 10.3389/fcimb.2023.1103957

**Published:** 2023-02-01

**Authors:** Pooja Sen, Lovely Gupta, Mukund Vijay, Maansi Vermani Sarin, Jata Shankar, Saif Hameed, Pooja Vijayaraghavan

**Affiliations:** ^1^ Anti-mycotic Drug Susceptibility Laboratory, Amity Institute of Biotechnology, Amity University Uttar Pradesh, Noida, India; ^2^ Department of Biotechnology and Bioinformatics, Jaypee University of Information Technology, Solan, India; ^3^ Amity Institute of Biotechnology, Amity University Haryana, Gurugram (Manesar), India

**Keywords:** Azole-resistant *Aspergillus fumigatus*, 4-allyl-2-methoxyphenol, biofilms, *MDR4*, *mfsC*

## Abstract

**Introduction:**

Antifungal therapy for aspergillosis is becoming problematic because of the toxicity of currently available drugs, biofilm formation on host surface, and increasing prevalence of azole resistance in *Aspergillus fumigatus*. Plants are rich source of bioactive molecules and antimicrobial activity of aromatic bioactive compounds draws attention because of its promising biological properties. The present study elucidated the antibiofilm activity of 4-allyl-2-methoxyphenol (eugenol) against azole-resistant environmental *A. fumigatus* isolates.

**Methods:**

Soil samples were collected from agricultural fields across India; azole-resistant *A. fumigatus* (ARAF) were isolated followed by their molecular identification. Antibiofilm activity of eugenol was calculated via tetrazolium based-MTT assay. The expression of the multidrug efflux pumps genes *MDR1, MDR4*, transporters of the *MFS* gene, *erg11A* gene encoding 14α demethylase, and transcription regulatory genes, *MedA*, *SomA* and *SrbA*, involved in biofilm formation of *A. fumigatus* were calculated by quantitative real time PCR.

**Results:**

Out of 89 *A. fumigatus* isolates, 10 were identified as azole resistant. Eugenol exhibited antibiofilm activity against ARAF isolates, ranging from 312 to 500 µg/mL. Confocal laser scanning microscopy analysis revealed absence of extracellular matrix of ARAF biofilm after eugenol treatment. The gene expression indicated significantly low expression of efflux pumps genes *MDR1*, *MDR4*, *erg11A* and *MedA* in eugenol treated ARAF isolates when compared with untreated isolates.

**Conclusions:**

Our results demonstrate that eugenol effects the expression of efflux pump and biofilm associated genes as well as inhibits biofilm formation in azole resistant isolates of *A. fumigatus*.

## 1 Introduction

*Aspergillus* infections manifest as chronic pulmonary aspergillosis, invasive aspergillosis, bronchitis, allergic bronchopulmonary aspergillosis and severe asthma with fungal sensitization. *Aspergillus* infections can cause life-threatening diseases in individuals with weakened immunity, having underlying diseases, or organ transplant (Denning et al., 2016). Invasive manifestation of aspergillosis is fatal, with mortality rate nearly 50% when treated promptly and >80% when the treatment is delayed (Kosmidis and Denning, 2015). *Aspergillus fumigatus*, a saprophytic pathogenic fungus, causes majority of pulmonary infections in immunocompromised patients (Bongomin et al., 2017). Azoles are currently recommended as first-line drugs to treat *A. fumigatus* infections. However, a steady global increase in azole resistance in *A. fumigatus*, resulting in therapeutic failures, has been a matter of serious clinical concern (Ahangarkani et al., 2020a). The resistant strains can be isolated from the patients taking azole drugs for a long time as well as from the environment because of prolonged usage of azole pesticides (Chowdhary et al., 2014; Ahangarkani et al., 2020b). Triazole resistance has been reported in azole naïve patients in India without previous triazole exposure, suggesting a possible role for environmental transmission of resistance (Dabas et al., 2018). Azoles target the *erg11A/cyp51A* gene; therefore, azole resistance due to mutation in this gene or its overexpression has been previously reported (Price et al., 2015). Several other resistance mechanisms such as overexpression of drug efflux pumps and transporters of the major facilitator superfamily (MFS) have also been reported (Da Silva Ferreira et al., 2004; Fraczek et al., 2013).

*A. fumigatus* also forms biofilms, thereby increasing the severity of fungal infection (Müller et al., 2011). Biofilm formation protects the fungus from host immune system and reduces the efficacy of antifungal drug therapy leading to development of antimicrobial resistance (Mowat et al., 2007). The minimal inhibitory concentrations (MICs) of antifungals, particularly azoles against *Aspergillus* biofilms are predominantly high (Beauvais and Latgé, 2015). *A. fumigatus* biofilms exhibit drug resistance due to biofilm-specific upregulation of efflux proteins and presence of an extracellular matrix (ECM) (Manavathu et al., 2014). Multidrug resistance (MDR) efflux pumps in *A. fumigatus* have been described and are associated with increased resistance to itraconazole (Nascimento et al., 2003; Da Silva Ferreira et al., 2004). MDR pumps transport a variety of toxic substrates such as azole drugs from the cells. Rajendran et al., 2011 reported the upregulation of the *MDR4* gene in *A. fumigatus* isolate and an *in vivo* mouse biofilm model after 24-h voriconazole treatment. This study suggested that the overexpression of the *MDR4* gene in *A. fumigatus* biofilm is associated with azole resistance. *A. fumigatus* regulatory protein *MedA* and transcription factor *SomA* are involved in biofilm formation and adherence (Gravelat et al., 2010). The disruption of the *MedA* gene leads to impairment of conidiation and biofilm formation. The *ΔmedA* mutant demonstrated reduced adherence to pulmonary epithelial and endothelial cells (Gravelat et al., 2010). Therefore, the treatment of infections caused by *Aspergillus* biofilms is a serious clinical challenge, emphasizing the need for novel and effective therapeutic agents.

The discovery of a novel antifungal and anti-biofilm agents with ideal features, such as broad-spectrum activity, low toxicity, minimal side effects, and enhanced bioavailability, is a key priority in medical mycology research (Brauer et al., 2019). Plants are extremely rich sources of bioactive molecules with analgesic, anti-inflammatory, anti-carcinogenic, anti-mutagenic, and even repellent properties (Pramod et al., 2010; Chung et al., 2014). 4-allyl-2-methoxyphenol, also known as eugenol, is a volatile naturally occurring phenolic bioactive constituent in clove oil, nutmeg, and basil (Ferreira et al., 2017). It possesses significant antioxidant, antifungal, and anti-inflammatory properties, in addition to analgesic and local anaesthetic activity (Huang et al., 2015; Marchese et al., 2017; Goswami et al., 2022). This compound offers a number of therapeutic benefits *via* inhibition of generative reactive oxygen and nitrogen species, scavenging of free radicals, and disruption of microbial biofilms (Khalil et al., 2017). Eugenol inhibits *Candida* spp. *via* altering fungal cell membrane and cell wall resulting in the release of cellular contents (Bennis et al., 2004; Ahmad et al., 2010). Inhibitory effect of eugenol has been observed in the initial phase of biofilm formation as well as in the mature biofilm of *Candida albicans* (Khan and Ahmad, 2012). However, inhibitory effect of eugenol on environmental isolates of azole-resistant *A. fumigatus* has not been reported to the best of our understanding. Therefore, the present study elucidated the antibiofilm activity of 4-allyl-2-methoxyphenol (eugenol) against azole-resistant environmental *A. fumigatus* isolates.

## 2 Materials and methods

### 2.1 Screening for azole-resistant *A. fumigatus* (ARAF) isolates from soil samples

A total of 110 soil samples were collected from various agricultural fields across India. Briefly, 2 g of each soil sample was suspended in 10 mL of sterile 0.9% normal saline, vortexed, and allowed to settle for 4-5 h. From this stock suspension, 100 μL of the supernatant was inoculated on Potato Dextrose Agar (PDA) plates supplemented with 0.5 mg/mL chloramphenicol and were incubated at 28 ± 2°C for 5 days. All samples were processed as biological triplicates. All *A. fumigatus* isolates grown on agar plates were identified by macroscopic and microscopic characteristics (de Hoog et al., 1995). *A. fumigatus* isolates were further screened for azole resistance by plating the spores on PDA containing (a) no drug as control; (b) 1 μg/mL itraconazole (ITC); (c) 1 μg/mL voriconazole (VRC), and (d) 0.5 μg/mL posaconazole (PSZ) (Tangwattanachuleeporn et al., 2017). The plates were incubated at 28 ± 2°C for 5 days. *A. fumigatus* isolates grown on azole-containing agar plates were further subcultured and maintained on PDA plates.

### 2.2 Molecular identification of ARAF isolates

Genomic DNA was extracted from the ten ARAF isolates using the cetyl trimethyl ammonium bromide (Lee et al., 1988; Wu et al., 2001). Molecular identification of the isolates as *A. fumigatus* was confirmed by the amplification and sequencing of full-length 18S internal transcribed spacer (ITS) region using the ITS1 (5’ TCC GTA GGT GAA CCT GCGG 3’) and ITS4 (5’ TCC TCC GCT TAT TGA TATGC 3’) primers (White et al., 1990) and partial sequencing of *β*-tubulin gene using Tub5 (5’ TGACCCAGCAGATGTT 3’) and Tub6 (5’ GTTGTTGGGAATCCACTC 3’) primers Mellado et al., 2007; Hoda et al., 2020).

The phylogenetic tree was prepared using *β-*tubulin gene sequence alignments in the MEGA (version11.0.13) program, using maximum likelihood method. The support for each clade was determined by bootstrap method with 1,000 replicates.

### 2.3 Antifungal susceptibility testing

The spores (conidia) of azole-resistant and wild-type *A. fumigatus* were harvested in sterile phosphate buffered saline (1× PBS) supplemented with 0.05% Tween 20; suspension was adjusted to 1×10^4^ conidia/mL in potato dextrose broth. MIC of ITC and VRC against ARAF with reference to wild-type strain of *A. fumigatus* (ATCC 46645) was determined using CLSI M38-A2 broth microdilution method (Alexander and Clinical and Laboratory Standards Institute, 2017) in a 96-well polystyrene plate (Tarsons, India). The experiment was carried out in triplicates. The stock of azole drugs was prepared in dimethyl sulfoxide. Two-fold dilutions were prepared in a 96-well microplate to obtain concentrations ranging from 64–0.125 µg/mL for ITC and VRC. Each well was inoculated with 100 µL of the conidial suspension except negative control. Microplate was incubated at 28 ± 2°C for 5 days, and the growth in each well was compared with that of the positive control. The MIC of a drug is determined as the lowest concentration with no visible growth relative to the drug-free control (Alexander and Clinical and Laboratory Standards Institute, 2017). The results were further analyzed with reference to epidemiological cutoff values (ECVs) for ITC: 1 μg/mL; VRC: 1 μg/mL (Pfaller et al., 2011; Lass-Flörl, 2014; CLSI, 2018).

### 2.4 *A. fumigatus* biofilm eradication activity of eugenol

The biofilm-eradication concentration (BEC_80_) of eugenol for a preformed fungal biofilm was calculated using MTT assay in a 96-well flat bottom microtiter plates with minor modifications (Sav et al., 2018). Conidial suspension (100 µL) in RPMI was added to each well of the flat bottom microtiter plate and incubated at 37 °C for 24 h without agitation. After 24 h, non-adherent conidia were removed by washing with 1× PBS. Thereafter, 100 μL of various concentrations of eugenol (2000–39 µg/mL) were added to the respective wells, and further incubated at 37 °C statically for an additional 24 h. Negative control wells contained only RPMI media. Wells with preformed biofilms without any treatment were considered as positive control. The effect of eugenol on the preformed fungal biofilms was estimated using a semi-quantitative viability based MTT reduction assay (Manavathu et al., 2014).

For confocal laser scanning microscopy (CLSM) analysis, the samples were processed as described by González-Ramírez et al. (2016) with minor modifications. The biofilms of ARAF isolates were cultured in a 12-well polystyrene plate at the calculated BEC_80_ of eugenol (Manavathu et al., 2014). Biofilm samples were stained with calcofluor white M2R (Sigma, St. Louis, MO, USA) and were analyzed using Nikon Instruments A1 Confocal Laser Microscope Series with NIS elements (C software, Japan). BEC_80_ is the concentration at which nearly 80% of fungal biofilm is eradicated.

### 2.5 Sequencing of the *cyp51A* gene

Two different primer sets (Tangwattanachuleeporn et al., 2017) were used for amplification of the *cyp51A* gene mutations (**Table 1**). Polymerase chain reaction (PCR) was performed using a final volume of 20 µL PCR buffer, containing 4 µL of fungal genomic DNA, 10 µL of PCR Master Mix (HiMedia, India), 0.50 µL (10 pmol/µL) of each primer and volume makeup by deionized water. Thermal cycling conditions for amplification was as follows: 95°C for 5 min followed by 35 cycles of denaturation at 95˚C for 30 s; annealing at 57.8˚C for 30 s and extension at 72˚C for 30 s; and final extension at 72˚C for 10 min. The amplified products were analyzed using agarose gel electrophoresis and purified using HiMedia quick gel purification kit according to the manufacturer’s instructions. Nucleotide sequencing was performed *via* Sanger’s sequencing using ABI 370 XL (Applied Biosystems). The sequence of the products was compared to the *A. fumigatus* wild-type *cyp51A* sequence accession no. AF338659 (Mellado et al., 2007; Rodriguez-Tudela et al., 2008) using the NCBI alignment service, Align Sequence Nucleotide Blast (https://blast.ncbi.nlm.nih.gov/Blast.cgi) and Clustal Omega tool (https://www.ebi.ac.uk/Tools/msa/clustalo/).

**Table 1:** Primers used for qRT-PCR.

S. No.	Gene name	Primer sequence (5ʹ-3ʹ)	Amplicon size (bp)	References
1.	*MedA*	F: GCC TTG CTA GGT AAGTTT GT R: TGT TCC TTC TGA ACC TCT C	348	Gupta et al., 2022
2.	*SomA*	F: ACG TGG CTA TCA TGA ATG TG R: TAC TGT CTC ACG TCG TTG CT	300	Gupta et al., 2022
3.	*SrbA*	F: TTG ATG CAG GAA GTA GAG GT R: AAA CTT CTC GGC TGT TAG TG	210	–
4.	*erg11A*	F: CACGTCAAGTCCCTATCTTC R: GTTTCAGGGACTCCTTTCTT	150	–
5.	*MDR1*	F: GCT TGG TGG AGC GCT TTT AC R: TCA TGG CCG TCC AGC AA	58	Sharma et al., 2019
6.	*MDR4*	F: TGG GAC TCG TCA TCT CAA C R: GGT GTG ACA AAC TGG AGG A	213	–
7.	*mfsC*	F: GGC AGT CCC GTC GCT CT R: CTT CGA CCT CGC GGA GAA	120	Sharma et al., 2019
8.	*β-tubulin*	F: TTG ACC CAG CAG ATG TTC G R: GGG AAT CCA CTC AAC GAA G	174	Hoda et al., 2020

### 2.6 Quantification of gene expression by qRT-PCR

Ten azole-resistant *A.fumigatus* isolates (eugenol-treated and untreated) along with a susceptible *A.fumigatus* (ATCC 46645; control) isolates were investigated for the expression analysis of the genes of interest by quantitative real time PCR (qRT-PCR; Gupta et al., 2022). Two micrograms of total RNA were reverse transcribed into first-stand cDNA using the Hi-cDNA synthesis kit (HiMedia, India) by following the manufacturer’s recommendations. Real-time PCR was performed using an ABI QuantStudio 3 (Applied Biosystems, Streetsville, Canada), and amplification products were detected with SYBR-green master mix (HiMedia, India) for fungal gene expression. The gene expression was estimated using the 2^− ΔΔCt^ method, with *β-*tubulin as the reference gene. The gene specific primers were designed for *MDR4, erg11A* and *SrbA* using Primer 3 software (http://primer3.ut.ee/) (Untergasser et al., 2012). The primer sets used in this study are listed in **Table 1**.

### 2.7 Statistical analysis

For biofilm percentage, statistical analysis was performed using non-linear regression in a dose response manner with 95% confidence interval (CI). The range of best fit value at 95% CI for all the ARAF isolates has been listed in **Table S1**. In gene expression analysis, one-way ANOVA test was used to analyse whether the expression levels were statistically different. The *p* value of ≤0.05 was considered significant. Statistical analyses were also performed using GraphPad Prism v8.0.2.263.

## 3 Results

### 3.1 Screening for ARAF isolates from environmental samples

We have isolated 245 *Aspergillus* isolates from 110 soil samples and were further identified on the basis of macroscopic and microscopic characteristics. Of these, 89 isolates were identified as *A. fumigatus* (**Table 2**). Agar screening assay revealed that 10 isolates were azole resistant.

**Table 2:** Environmental ARAF isolates (identified macroscopically and microscopically) obtained from the soil samples collected from different regions of India.

S. No.	Sites	Total number of soil samples collected	Number of *Aspergillus* isolates	Number of *A. fumigatus* isolates	Number of ARAF isolates
1.	Uttar Pradesh	35	45	34	8
2.	Madhya Pradesh	7	33	4	–
3.	Uttaranchal	6	20	5	–
4.	Assam	9	19	3	–
5.	Bihar	18	30	5	–
6.	Rajasthan	16	57	32	2
7.	Haryana	9	31	2	–
8.	West Bengal	2	–	–	–
9.	Delhi	5	–	–	–
10.	Punjab	3	10	4	–
	**Total**	110	245	89	10

(-): absent.

### 3.2 Molecular identification of ARAF isolates

The 18S ITS region and *β*-tubulin gene sequence were amplified using PCR and visualized using ethidium bromide staining on agarose gel. The amplified regions of ITS and *β*-tubulin gene were further sequenced. The sequences of ten ARAF isolates were 99%–100% identical to the *A. fumigatus* sequence in the NCBI database. The identified fungal 18S ITS sequences were submitted to GenBank NCBI databases (https://www.ncbi.nlm.nih.gov/). The GenBank accession numbers generated for the submitted ITS sequences are MW485771, MW045410, MT941034, MW045442, MW045443, MW045444, MT941029, MZ284948, MZ379445, MZ379446 and for the *β*-tubulin gene sequences are ON759330, ON792384, ON792385, ON792386, ON792387, ON792388, ON792389, OP891221, ON792391 and OP891220. Phylogenetic tree of partial *β*-tubulin gene sequence by maximum likelihood was depicted in **Figure S1**.

### 3.3 Antifungal susceptibility testing

The results of antifungal activity of azole drugs against environmental isolates of ARAF are presented in **Table 3**. MIC of 9/10 isolates against ITC were above its ECV i.e., >1 μg/mL whereas three isolates had MIC >1 μg/mL against VRC (**Table 3**). The wild-type strain of *A. fumigatus* ATCC 46645 was sensitive to the azoles.

**Table 3:** Antifungal susceptibility profile of 10 A*. fumigatus* isolates against azoles.

Isolate	MIC (µg/mL)	
	ITC	VCZ
B15	**4**	0.5
RT28	**2**	0.125
B08	**16**	0.125
Y29	**2**	0.125
B19	0.125	**2**
RB01	**2**	0.125
B03	**8**	**2**
OF26	**8**	**2**
R01	**4**	0.125
R03	**4**	0.125
Wild-type ATCC 46645	0.125	0.125

*Bold letters depicted MIC above ECV values i.e.,>1 µg/mL for itraconazole (ITC) and voriconazole (VCZ).

### 3.4 *A. fumigatus* biofilm eradication

**Figure 1:**
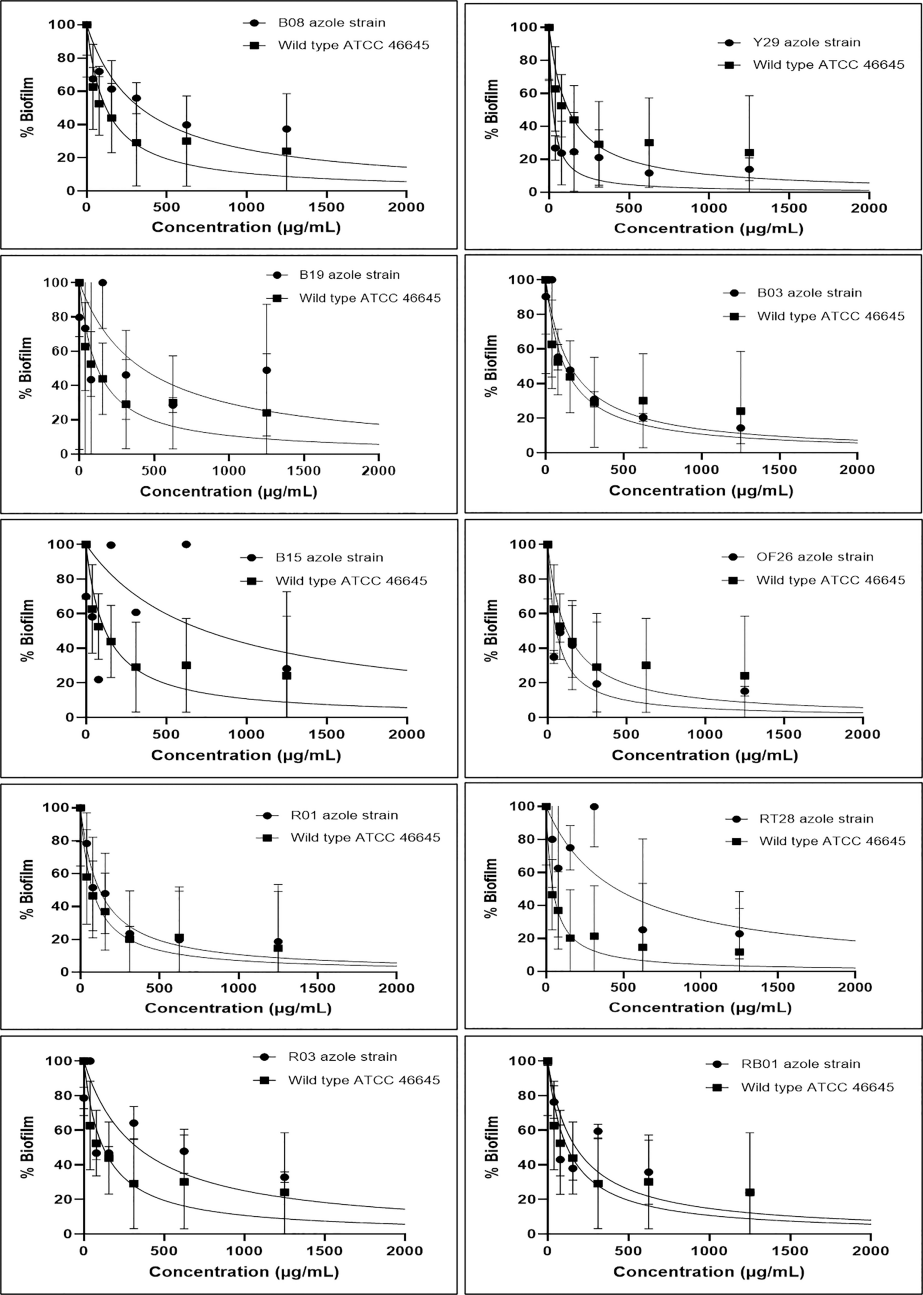
Effect of eugenol on biofilm percentage in azole resistant (B08, Y29, B19, B03, B15, OF26, R01, RT28, R03 and RB01) isolates and susceptible (wild-type ATCC 46645). BEC_80_ of eugenol ranged from 312–500 µg/mL except B19, B15, and RT28 isolates having BEC_80_ of >1000 µg/mL. Experiment was carried out in duplicate and analysed for statistical significance using non-linear regression in a dose response manner with 95% confidence interval (CI).

The effect of eugenol on the biofilms formed by ARAF isolates as well as wild-type *A. fumigatus* ATCC 46645 strain was evaluated using semi-quantitative approach *via* MTT assay. The graphs depicted the percentage of biofilm at different concentrations of eugenol in comparison to wild type ATCC 46645 (**Figure 1**). The effective concentration of eugenol to eradicate the fungal biofilm was calculated as 312–500 µg/mL. In this range, biofilm eradication was observed up to 80% in a dose-responsive manner with statistical significance (95% CI). The BEC_80_ of eugenol for the biofilm of azole-resistant isolates was similar to that of wild-type ATCC 46645. In contrast, B19, B15, and RT28 isolates showed BEC_80_ of >1000 µg/mL.

**Figure 2:**
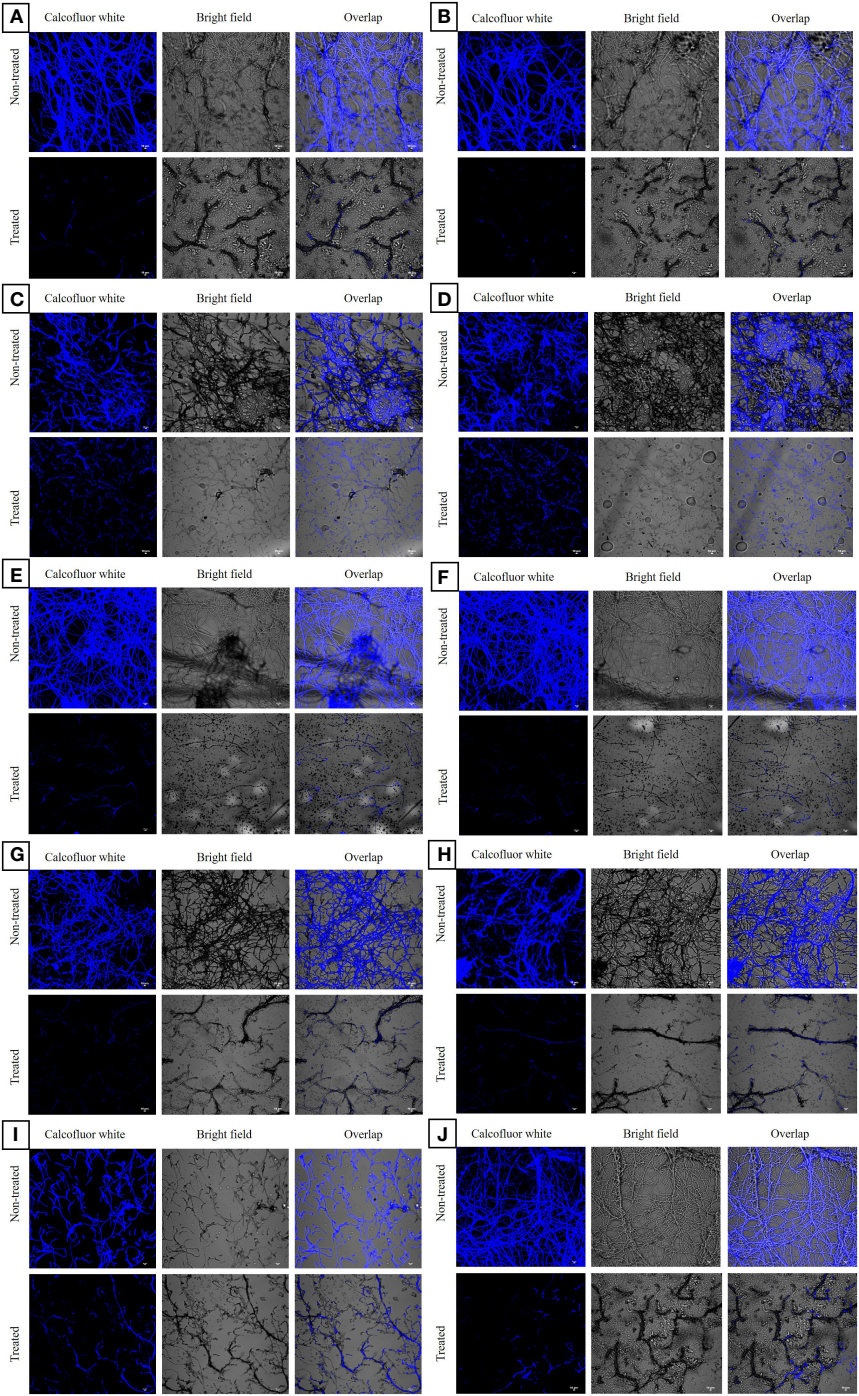
Confocal laser scanning microscopic images of biofilms of azole-resistant *A. fumigatus* isolates stained with calcofluor white dye. It depicted eugenol untreated and treated azole-resistant **(A-J)** B08, Y29, RT28, B15, RB01, B03, OF26, R01, B19 and R03 isolates, respectively. In eugenol-treated samples disintegrated hyphae without ECM were observed at 20 × magnification. The chitin component is stained with the calcofluor white dye and shown in three images with and without fluorochrome and overlapping. Scale Bar: 10 μm.

CLSM analysis of the untreated azole-resistant isolates and wild type ATCC 46645 showed aggregated multicellular hyphae enclosed within ECM. All the ARAF isolates indicated similar biofilm morphology as of wild type ATCC 46645. In untreated azole-resistant isolates, highly compact intermingled hyphae were observed, thereby strengthening the structure to reduce the effect of antifungals. When the susceptible and ARAF isolates were treated with eugenol at BEC_80,_ we observed that the eugenol effectively eradicated the ECM and hyphae disintegrated in seven ARAF isolates (B08, Y29, RB01, B03, OF26, R01, and R03) (**Figure 2**). However, B19, B15, and RT28 isolates showed comparatively less eradication of biofilm when treated with eugenol at BEC_80_.

### 3.5 Mutation analysis of the *cyp51A* gene

**Figure 3:**
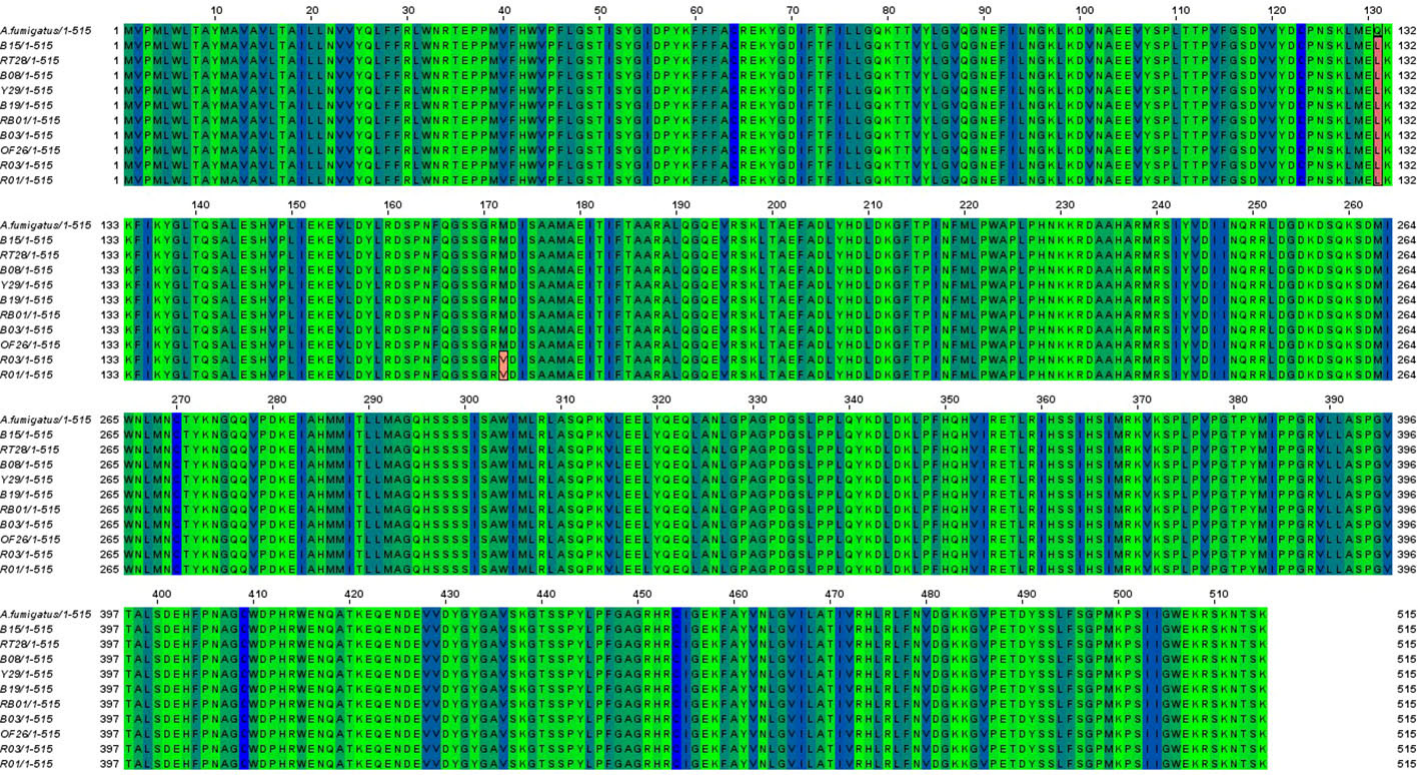
Sequence alignment of Cyp51A proteins from susceptible *A. fumigatus* (accession no. AF338659) and azole resistant isolates (B08, Y29, B19, B03, B15, OF26, R01, RT28, R03 and RB01). Identical residues were marked in blue and green shade whereas amino acid substitutions found in the Cyp51A sequence of the ARAF isolates were highlighted in red color.

The *cyp51A* gene of 10 ARAF isolates was sequenced and aligned with that of susceptible *A. fumigatus* strain accession no. AF338659 to identify non-synonymous mutations (**Figure 3**). Amino acid alterations were observed as a result of nucleotide substitutions in the *cyp51A* gene. The amino acid change Q131L was detected in all resistant environmental isolates. Two ITC-resistant isolates (R01 and R03) were found to have M172V non-synonymous nucleotide mutation in the *cyp51A* region. Some other reported mutations were not found in our tested isolates.

### 3.6 Gene expression analysis

**Figure 4:**
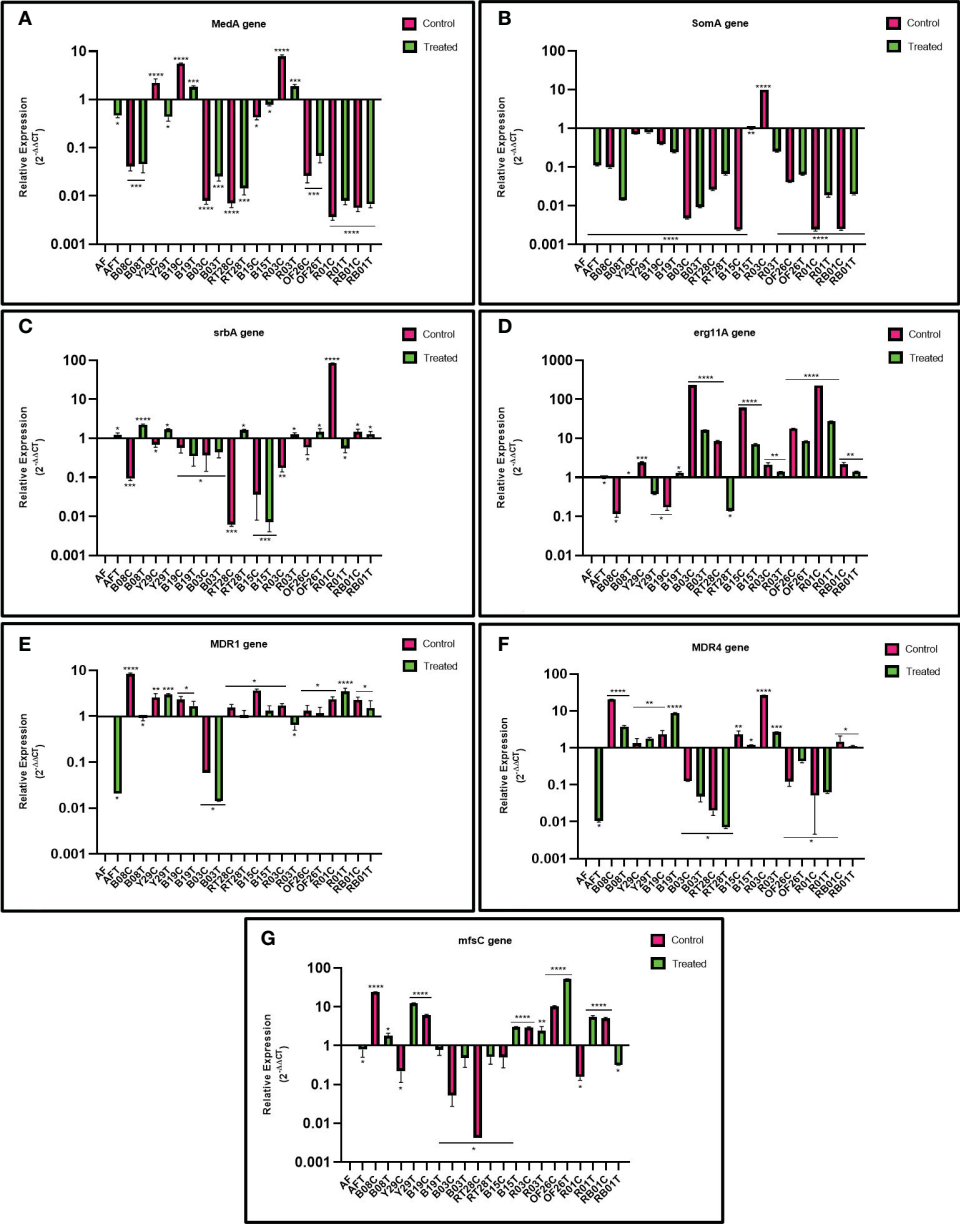
Relative expression of *MedA* **(A)** *SomA* **(B)** *SrbA* **(C)** *erg11A* **(D)** *MDR1* **(E)** *MDR4* **(F)** and *mfsC* **(G)** genes in azole-resistant environmental isolates relative to that in susceptible *A fumigatus* (wild type ATCC 46645) in untreated and eugenol-treated conditions. *β*-tubulin was used as a housekeeping gene to normalize transcription levels.

The impact of eugenol treatment on MDR efflux pump genes *MDR1* and *MDR4*, transporters of the MFS gene *mfsC*, sterol 14α-demethylase encoding gene *erg11A*, and transcription regulatory genes, *MedA, SomA* and *SrbA*, involved in biofilm formation of *A. fumigatus* were investigated by reverse transcription followed by qRT-PCR for differential gene expression. **Figure 4** demonstrates the two-fold relative expression of gene of interest in eugenol-treated and untreated ARAF isolates compared with the susceptible *A. fumigatus* (wild type ATCC 46645).

The *MedA* gene was upregulated in 3/10 untreated ARAF isolates compared to susceptible *A. fumigatus*. Except R03 untreated isolate, none of the ARAF isolates showed upregulation of the *SomA* gene. Expression analysis of the *SrbA* gene revealed that the gene is downregulated in both conditions, as was observed in three (B19, B03, B15) out of ten isolates. Relative expression of gene *MDR4* showed upregulation in six ARAF untreated isolates. Expression was decreased after eugenol treatment in four isolates (B08, B15, R03 and RB01). The *MDR1* gene was upregulated in all untreated isolates except B03 and expression was decreased after eugenol treatment in seven isolates. The *erg11A* gene was highly expressed in all the treated as well as untreated ARAF isolates except Y29, RT28 (treated) and B19, B08 (untreated). *mfsC* gene expression analysis was downregulated in two treated isolates (B19 and RB01).

## 4 Discussion

Environmental surveillance studies of azole resistance have been reported from many countries including Italy, Austria, India, Thailand, and the USA (Mortensen et al., 2010; Chowdhary et al., 2014; Tangwattanachuleeporn et al., 2017). However, the global prevalence of ARAF is still not clearly determined in routine resistance testing. Additionally, failure of current mainstay antifungals is a serious concern for clinicians as well as researchers. As azoles are the frontline antifungals, resistance could complicate the management of chronic and invasive diseases caused by *A. fumigatus* leading to treatment failure.

In the present study, the prevalence of ARAF in the Indian environment was 11.23% which is lower than that reported from European countries (Talbot et al., 2018) but higher than that reported from most of the Asian nations (Tangwattanachuleeporn et al., 2017). The management of azole-resistant aspergillosis remains a challenge and there are no guidelines as well as appropriate recommendations for this purpose. Understanding local rates of environmental resistance is critical for efficient patient management and it has been proposed that the level of environmental *A. fumigatus* azole resistance should be used to guide treatment of *Aspergillus* infections (Snelders et al., 2011). Triazole antifungals are crucial for the treatment and prophylaxis of infections occurring due to *Aspergillus* spp. The main route of azole resistance developed in the environment is related to the inappropriate use of azole-based pesticides. Azole pesticides have similar molecular structure as that of triazole drugs and resistant strains that develop in the environment are more efficient for developing cross resistance towards medical triazoles (Snelders et al., 2012). The clinical studies suggest that liposomal amphotericin B or a combination of VRC or PSZ with an echinocandin may be effective treatment (Elefanti et al., 2013). Eugenol, being a phytochemical, has already been reported to have antifungal activity in *Candida* spp. The MIC of eugenol have been reported in the range of 375–750 µg/mL against *Candida dubliniensis* and *Candida tropicalis* and 200–400 µg/mL against *Candida krusei* (De Paula et al., 2014; Sharifzadeh and Shokri, 2021). Eugenol containing essential oils were found to transform the hyphal ultrasound and virulence factors of *A. fumigatus* and *Trichophyton rubrum* (Gayoso et al., 2005). Antifungal activity of eugenol against *Fusarium oxysporum*, *Lycopersici* 1322 has also been reported, suggesting that eugenol can be used in preventive and therapeutic applications (Park et al., 2009).

Biofilm-associated infections have very high mortality rate and difficult to cure with existing drug therapies. Mowat et al. (2007) reported the formation of biofilm structures in *A. fumigatus* cultures which were resistant to antifungal drugs. Eugenol exhibited significantly favourable antifungal activity on various preformed biofilms, adherent cells, subsequent biofilm formation, and cell morphogenesis of *C. albicans*, without instigating haemolytic activity in human erythrocytes (Palaniappan and Holley, 2010). Similar results have been reported by De Paula et al. (2014) against *Candida* spp. In the present study, antibiofilm activity of eugenol against environmental isolates of ARAF suggested that effective BEC_80_ range of eugenol was 312–500 µg/mL against seven ARAF isolates and wild-type azole-susceptible *A. fumigatus* strain. Eugenol eradicated fungal biofilm along with the elimination of ECM in a dose-dependent manner. Further, the CLSM analysis supported the results of MTT assay with eradication of *A. fumigatus* biofilm. The biofilms of three ARAF isolates (B19, B15, and RT28) were less affected by eugenol treatment. CLSM analysis of eugenol-treated ARAF isolates revealed eradication of ECM and intermingled hyphae except B19 isolate.

Azoles target 14α-demethylase encoding *erg11A* gene and block ergosterol biosynthesis (Price et al., 2015). The overexpression of this gene in ARAF isolates could be a possible drug resistance mechanism. The present study calculated the expression of *erg11A* gene with and without eugenol; the gene was significantly overexpressed in six ARAF isolates under both conditions whereas it was found to be downregulated in two isolates (Y29 and RT28) after eugenol treatment. Ergosterol biosynthesis gene expression increased in biofilms formed by planktonic cultures. In azole resistant strains *cyp51A* gene plays a crucial role, since the deletion of the *cyp51A* gene resulted in increased sensitivity to azoles (Garcia-Rubio et al., 2018). Sterol biosynthesis is directly impacted by iron and oxygen levels and reductions in these key molecules stimulates ergosterol biosynthesis gene expression through *SrbA* dependent mechanisms (Blatzer et al., 2011). In submerged biofilm cultures, oxygen levels are depleted over the course of biofilm development and correspond with an increase in transcription of the *SrbA* dependent gene *erg25A* (Kowalski et al., 2020). Thus, as biofilm cell density increases, oxygen levels within the immature biofilm become depleted, leading to both an increase in expression of ergosterol biosynthetic genes and a decrease in metabolism. These two factors may contribute to the localized increase in antifungal resistance within the developing biofilm. Expression analysis of the *SrbA* gene revealed the downregulation of gene in the presence and absence of eugenol in the following three ARAF isolates B19, B03, B15 and also in untreated ARAF isolates encoded B08, Y29, RT28, and OF26. The role of overexpression of efflux pumps, ATP-binding cassette (ABC) transporters and transporters of the MFS gene, in *C. albicans* and *C. glabrata* has been well documented (Cannon et al., 2009; Morschhäuser, 2010). Two major facilitator transporters, AfuMDR1 and AfuMDR2, were identified in *A. fumigatus* by Tobin et al. (1997). Nascimento et al. (2003) showed upregulation of two transporters, *MDR3* and *MDR4* in ITC resistant *A. fumigatus* strains, the latter of which has also been shown to be upregulated in a biofilm phenotype. Overexpression of efflux pumps in azole resistance has also been emphasized in other studies (Da Silva Ferreira et al., 2004; Fraczek et al., 2013). The relative expression of the MDR efflux pumps genes *MDR1* and *MDR4* was analyzed in this study, and we observed that *MDR1* was upregulated in nine isolates except B03. After eugenol treatment, the relative gene expression decreased in two ARAF isolates (B03 and R03). *MDR4* gene is reported in *C. albicans* biofilms and is associated with drug detoxification (Mukherjee et al., 2003). Overexpression of *MDR4* gene in azole resistance *A. fumigatus* strains after 24-h VRC treatment has been reported by Rajendran et al. (2011). Upregulation of *MDR4* was analyzed in six untreated isolates and after eugenol decreased in four ARAF (B03, OF26, R01 and RT28) isolates. But overexpression of the gene after eugenol treatment when compared to untreated has not been observed in our study. MDR pumps in *A.fumigatus* are associated with triazole resistance (Rajendran et al., 2011). In our study, the overexpression of the *MDR4* gene suggested that this gene is linked with ITC resistance and after eugenol treatment gene expression was decreased. Further, transcription regulatory genes *MedA* and *SomA* were downregulated in all the eugenol-treated ARAF isolates except B19 and R03 for *MedA* gene expression and similar results were reported by Gupta et al. (2022) for the downregulation of *MedA* and *SomA* genes in presence of isoeugenol. Further, *mfsC* gene expression analysis showed that the gene was downregulated in the four treated ARAF isolates (B19, RT28, B03 and RB01). Our observations suggest that many unidentified molecular mechanisms are associated to biofilm formation in environmental ARAF isolates which still needs to be explored. Further investigation of the relationship between the eugenol and MDR efflux pump genes and their pathways in *A.fumigatus* virulence and azole susceptibility may be needed to understand the mechanism of the observed interactions.

We observed notable antibiofilm activity of 4-allyl-2-methoxyphenol (eugenol) against azole-resistant *A. fumigatus* isolates. This activity appears to be mediated in some ARAF isolates *via* the inhibition of the *MedA* and efflux pump MDR genes. The use of eugenol alone or in combination with conventional antifungal drugs could be a valuable addition to current therapeutic strategies for treating azole-resistant *A fumigatus* infections.

## Data availability statement

The datasets presented in this study can be found in online repositories. The names of the repository/repositories and accession number(s) can be found in the article/**Supplementary Material**.

## Author contributions

PS and LG performed literature search, conducted all the experiments, and drafted the manuscript. MV also performed experiments and helped in manuscript editing. SH analysed transcriptional data. MS and JS critically reviewed the manuscript and corrected and PV conceptualized the idea as well as critically analysed the manuscript. All authors contributed to the article and approved the submitted version.
