# Supplementary material for: 4-Allyl-2-methoxyphenol modulates the expression of genes involved in efflux pump, biofilm formation and sterol biosynthesis in azole resistant Aspergillus fumigatus
Source: Front Cell Infect Microbiol. 2023 Feb 1;13:1103957. doi: 10.3389/fcimb.2023.1103957 (PMC9929553; doi:10.3389/fcimb.2023.1103957)
Supplement: Supplementary file 1 [file DataSheet_1.docx]

**Supporting document S1**

**Table S1.** The range of best fit value at 95% confidential interval for all the ARAF isolates using non-linear regression in a dose response manner via GraphPad Prism software version 8.0.2.263**.**

| **ARAF isolate** | **95% CI (profile likelihood)** | **Range of CI (Best fit value)** |
| --- | --- | --- |
| RT28 | logIC50 | 1.922 to 3.374 |
| B19 | logIC50 | 1.939 to 3.294 |
| R01 | logIC50 | 1.818 to 2.338 |
| OF26 | logIC50 | 1.314 to 2.094 |
| B15 | logIC50 | 2.308 to 3.368 |
| Y29 | logIC50 | 0.9612 to 1.695 |
| B03 | logIC50 | 1.904 to 2.489 |
| R03 | logIC50 | 2.223 to 2.820 |
| B08 | logIC50 | 2.336 to 2.715 |
| RB01 | logIC50 | 1.954 to 2.527 |

**
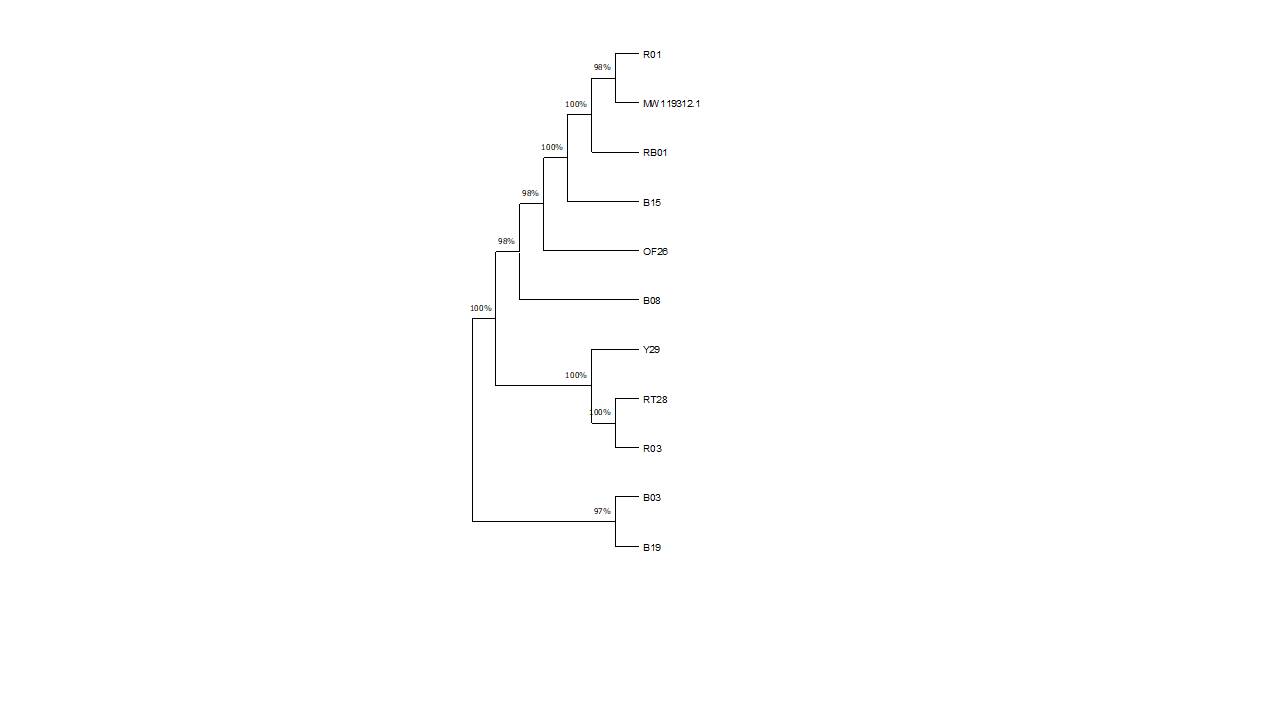
**

**Figure S1.** The phylogenetic tree was prepared using *β*-tubulin gene sequence alignment in the MEGA (version11.0.13) program, using maximum likelihood method. The phylogenetic tree revealed that the fungal isolates shared one common ancestor and they formed one main cluster (clade) which divided to form three subclade supported by 98%, 100% and 97% site coverage. Tree revealed that the R01, MW119312.1, RB01, B15, OF26, and B08 formed the first sub-clade and Y29, RT28, and R03 formed second and sub-clade third consist of B03 and B19. The isolates R01, RB01, B15, OF26, and B08 are showing a close relationship with MW119312.1 (GenBank sequence).
